# Immune organoids: emerging platforms for modeling and analyzing human adaptive immunity

**DOI:** 10.3389/fimmu.2025.1632117

**Published:** 2025-08-06

**Authors:** Tianlong Li, Dingkun Peng, Meng Yao, Meilin Li, Yijing Wang, Su Li, Ding Zhang, Bo Yang, Hua-Ji Qiu, Lian-Feng Li

**Affiliations:** ^1^ State Key Laboratory for Animal Disease Control and Prevention, Harbin Veterinary Research Institute, Chinese Academy of Agricultural Sciences, Harbin, Heilongjiang, China; ^2^ College of Veterinary Medicine, Shanxi Agricultural University, Taigu, Shanxi, China

**Keywords:** immune organoids, ex vivo culture, microfluidic chips, engineered tissues, immune organs

## Abstract

The rapid advancement of vaccines and immunotherapies has significantly improved public health. However, a significant translational gap remains between basic research and clinical application, largely attributed to the disconnect between *in vitro* studies and *in vivo* models. To bridge this gap, *in vitro* models of immune organs, including bone marrow, thymus, spleen, lymph nodes, and tonsils, have emerged as a promising solution. By integrating cutting-edge technologies such as *ex vivo* culture, microfluidic chips, engineered tissues, and organoid models, researchers have successfully established a new-generation *in vitro* immune simulation platform. This review systematically summarizes recent progress in immune organ-based *in vitro* models, outlines the current technological landscape and highlights the unique advantages of immune organoids within this field. Notably, we classify immune organoids into strictly and broadly defined categories based on their origin and construction methodology, while emphasizing the importance of multi-model integration. This platform provides a novel framework for advancing translational immunology research, particularly in the fields of adaptive immunity and vaccine development.

## 1 Introduction

The successful development of mRNA vaccines during the COVID-19 pandemic has further advanced immunology research (1). The pandemic has shown that infectious diseases remain a global threat (2, 3), with persistent infectious pathogens and new epidemic risks, which highlights the need to explore disease mechanisms for better prevention and treatment strategies (4). This phenomenon is influenced by various environmental and host factors, such as pollution and aging, among which immune cell aging is particularly significant. The impacts of these factors on human immune functions deserve in-depth investigation (5, 6).

In human cell research, two-dimensional cultured cell lines remain the primary focus. These classical cell lines are cost-effective, easy to manipulate, and compatible with various experimental techniques. However, establishing these cell lines is inefficient, and extensive genetic and phenotypic alterations occur under culture conditions (7, 8). Currently, research predominantly focuses on *in vivo* immune responses, including cell engineering, vaccine development, and immunotherapies (9, 10). Since most human data are confined to cells and molecules found in the blood, our understanding of immunity at the tissue level mainly comes from *in vivo*, *ex vivo*, and *in vitro* immune models.

Laboratory model systems are essential for elucidating disease mechanisms, with *in vivo*, *in vitro* and *ex vivo* models serving as the foundation of our understanding of infectious diseases (11). *Ex vivo* culture methods, including explants and slices, bridge the gap between *in vivo* animal models and *in vitro* cell cultures (12, 13). These tissue samples preserve the original structural and functional characteristics of organs while enhancing experimental operability. However, removing an organ from its natural environment disrupts physiological fluid flow dynamics. Microfluidic chip systems dynamically regulate the introduction of antigens, cells, and other elements (such as cytokines or growth factors) (14, 15). By introducing fluid flow and controlled microenvironments under *ex vivo* and *in vitro* conditions, microfluidic chips serve as versatile platforms for studying immune responses. Nevertheless, many chips are customized for specific functions, requiring tailored designs for different biological problems. Engineered tissues provide highly flexible platform for investigating the functions of various immune cells (14, 16). By constructing model tissues from scratch, researchers can explore specific cell-cell and cell-matrix interactions under tightly controlled conditions. However, most models rely on immortalized cell lines, lacking the diversity of primary cells. Although technologies such as *ex vivo* cultures, microfluidic chips, and engineered tissues exhibit certain advantages over animal models, they also have inherent limitations. Current systems fail to fully recapitulate the multicellular architecture of immune organs, thereby limiting the study of regulatory mechanisms in the adaptive immune system.

Organoids are three-dimensional (3D) multicellular self-organizing tissues that mimic the structure, function, and complexity of their corresponding internal organs (17, 18). Over the past five years, immune organoids have been developed and utilized to advance immunological research (19–22). They are primarily derived from lymphoid tissues, including the bone marrow, thymus, spleen, lymph nodes (LNs), and tonsils (21, 23, 24),. Immune organoids hold significant potential to overcome the limitations of animal models, thereby advancing human immunology research (25). For example, bone marrow organoids derived from pluripotent stem cells encompass diverse cell types and exhibit vascularized architectures that faithfully replicates the bone marrow’s physiological microenvironment (26). Similarly, tonsil organoids crafted from discarded tonsil tissue following tonsillectomy demonstrate remarkable capabilities in mimicking germinal center attributes, including somatic hypermutation (SHM), antigen-specific antibody production, affinity maturation, and class switching (22). This innovative technology represents a significant breakthrough, enabling the development of physiologically relevant adaptive immune system models and addressing certain constraints of alternative clinical methodologies.

Despite considerable advancements in immunological research, many intricate mechanisms of the immune system remain poorly understood. Additionally, the development of relevant *in vitro* models remains insufficient or nonexistent. This review systematically examines the critical roles of key immune organs models, including the bone marrow, thymus, spleen, LNs, and tonsils, in human adaptive immunity, integrating recent technological advances with immunological insights to demonstrate their translational applications (**Table 1**). Although other immune-associated tissues such as Peyer’s patches and the appendix also contribute to mucosal immunity, their corresponding models remain underdeveloped and are therefore outside the scope of this review, primarily due to limited research progress and technical challenges in replicating their specialized microenvironments.

**Table 1:** Comparison between traditional and advanced immune models.

Models	Advantages	Disadvantages
Animal models	Physiological complexity, immune cell diversity (27)	Species differences, ethical issues, high costs, individual variability (27, 28)
Slices	Original structure retention, simple operation, short-term experiment suitability (12, 29)	Short survival time, low reproducibility, poor feasibility (12)
Microfluidic chips	High-throughput screening capability, high operability, high reproducibility (30, 31)	Lack of biological sample repositories, poor immune response simulation (30, 31)
Engineered tissues	Genome editing capability, high-throughput screening capability, high operability (12, 32)	Functional limitations, low reproducibility (12)
Immune organoids	Highly bionic, individualized model, long term culture feasibility, animal experiment reductions (22, 33)	Incomplete functionality, technological complexity, high costs, standardization difficulty (25, 34)

## 2 Immune organs and tissues: indispensable systems for immunity homeostasis

The physiological functions of immune organs are fundamentally determined by their specialized tissue architectures and cellular ecosystems. These structural and compositional features create unique microenvironments that directly influence immune cell development, activation, and functional responses. Below we systematically examine both the physiological roles of major immune organs and the tissue-level organization enabling these functions.

### 2.1 Physiological functions of immune organs and tissues

As the body’s primary defense mechanism against foreign pathogens, the immune system plays an indispensable role in maintaining health. Within this intricate defense network, the adaptive immune response, primarily mediated by T and B cells, constitutes a pivotal mechanism for eliminating infections. Naive T cells become activated upon recognition and binding to their specific antigens, subsequently undergoing a series of complex yet orderly differentiation and proliferation processes that result in effector immune responses (35). In the context of antibody-mediated humoral immunity, follicular helper T cells, a specialized subset of antigen-specific T cells, facilitate the maturation of B cells through intimate interactions (36, 37), leading to the differentiation of B cells into memory B cells or plasma cells capable of secreting antibodies. Once this activation cascade is initiated, T cells and B cells traverse the circulatory system to various peripheral tissues, primed to respond swiftly to potential threats (12, 35, 38). Following the resolution of the initial threat, these activated memory cells do not dissipate; instead, they persist in infected tissues, LNs, or bone marrow, forming an immune reservoir that can be rapidly reactivated upon encountering the same or similar pathogens (39–42), thereby ensuring long-term and effective immunity against previously encountered pathogens.

### 2.2 Immune cell development of distinct organs

Immune cell development begins in the bone marrow, where T cell precursors migrate to the thymus for further differentiation, while B cells, natural killer cells, and myeloid cells, including macrophages and dendritic cells (DCs), complete their development in the bone marrow and then migrate to secondary lymphoid organs (SLOs), such as LNs and the spleen (12). In SLOs, immune cells are activated in response to specific pathogens or molecules and then migrate back into the bloodstream and sites of infection, cancer, or disease. These migration patterns suggest that immune cells programmed in different organs, such as spleen or LNs, can migrate throughout the host to initiate systemic and specific responses (16, 43).

**Figure 1:**
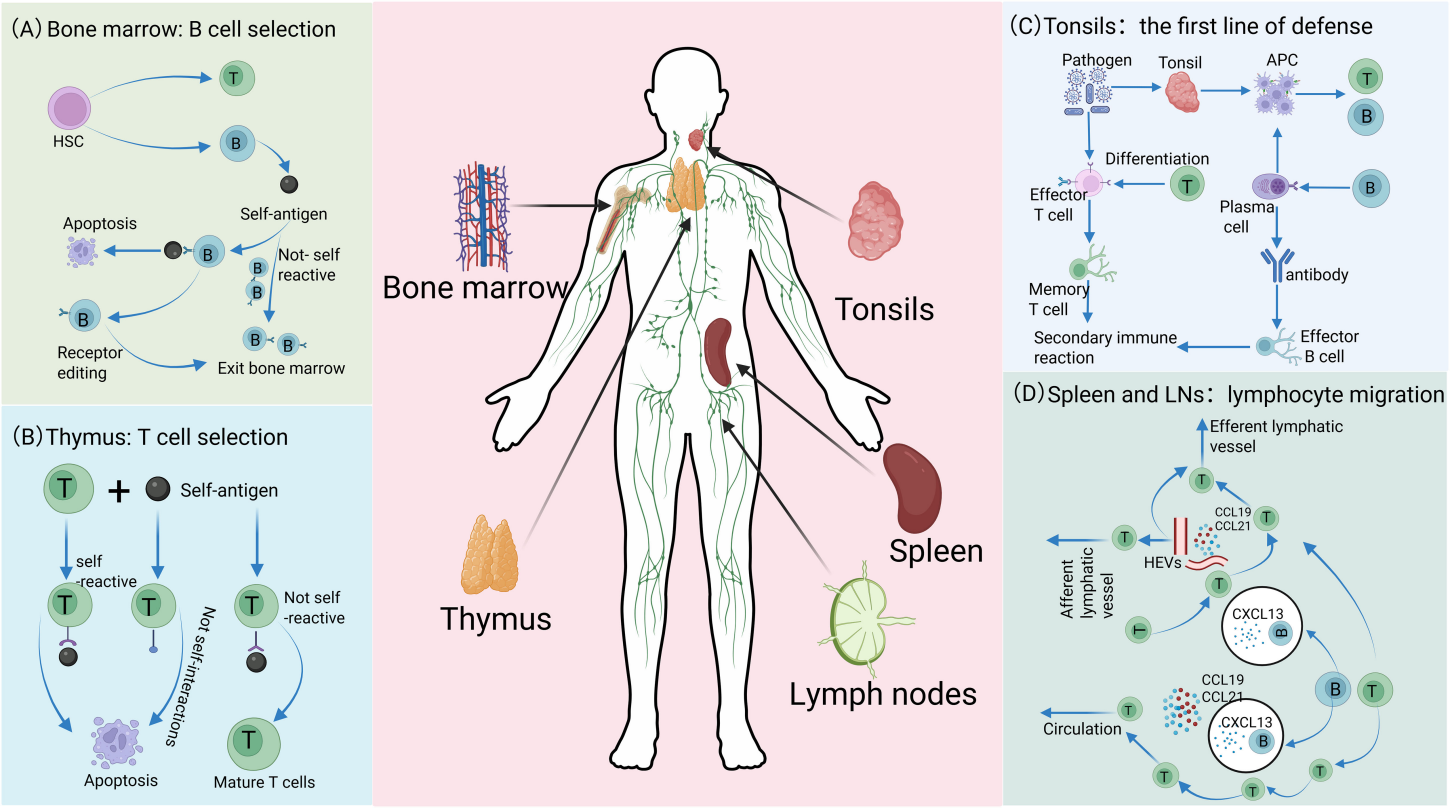
Distinct roles of immune organs in cellular development. **(A)** In the bone marrow, lymphocytes originate from HSCs. Among these, the majority of B cells matures and migrates to SLOs, while a small subset exhibiting high self-antigen affinity is eliminated through clonal deletion or receptor editing, thereby ensuring the maintenance of autoimmune tolerance. **(B)** After T cell precursors are generated in the bone marrow, they migrate to the thymus for maturation and selection. T cells with strong self-antigen binding or ineffective interaction with self-molecules are eliminated, whereas non-self-reactive precursors successfully mature and differentiate into functional T cells. **(C)** The tonsil serves as the first line of defense, capturing pathogens. Its APCs process antigens and activate T and B cells, promoting T cell differentiation into effector and memory T cells, and B cell differentiation into plasma and memory B cells. Plasma cells secrete antibodies, effector T cells eliminate pathogens, and memory cells establish immune memory to enable robust secondary responses. **(D)** Lymphocytes enter SLOs through distinct migration mechanisms and achieve region-specific localization. In the spleen, lymphocytes access the white pulp *via* MZ, with migration precisely regulated by chemokine receptor signaling: B cells migrate to follicles guided by CXCL13, while T cells are recruited to T cell zones in response to CCL19 and CCL21. Although the mechanisms of lymphocyte egress from the white pulp remain incompletely understood, this process is critical for maintaining systemic immune homeostasis. In LNs, lymphocytes primarily enter through HEVs rather than afferent lymphatic vessels. Guided by CXCL13, CCL19, and CCL21, they home to B cell follicles or T cell zones, respectively, and eventually exit *via* efferent lymphatics to re-enter systemic circulation.

In the bone marrow, hematopoietic stem cells (HSCs) develop into common precursor cells that differentiate into immune cells. Myeloid precursors give rise to APCs, including macrophages and DCs, which are responsible for detecting foreign molecules in blood and tissues (16, 44). Simultaneously, the bone marrow, as the center of proliferation and differentiation of HSCs, not only supports the entire maturation process of B cells but also produces T cell precursor cells (**Figure 1A**). These T cell precursors then migrate to the thymus and undergo rigorous positive selection (ensuring functional T cell receptor expression) and negative selection (clearing autoreactive T cells), ultimately forming a mature T cell repertoire with appropriate antigen specificity and affinity (12, 45, 46) (**Figure 1B**). These processes ensure that T cells express relevant molecules and integrate the corresponding signaling mechanisms required for immune signals from APCs. Meanwhile, through rigorous screening and regulation, they avoid inappropriate binding to autoantigens, thereby preventing abnormal responses that may lead to autoimmune diseases (47, 48). The spleen, LNs, and MALT, including the tonsils, constitute the principal sLOs where lymphocytes execute their immunological functions. These strategically distributed organs function as an integrated filtration network, systematically screening and processing the molecular and cellular constituents of extracellular fluids, including lymph, blood and interstitial fluid, thereby orchestrating targeted immune responses against potential pathogens (49, 50). The tonsils, as crucial components of MALT, play pivotal roles in orchestrating mucosal immune responses (51). Their distinctive crypt architecture, characterized by deep invaginations of the epithelial surface, creates an optimized microenvironment for efficient antigen capture, processing, and presentation, thereby facilitating the initiation of robust immune responses against respiratory and gastrointestinal pathogens (51–53). Within this specialized lymphoid structure, B lymphocytes undergo antigen-driven differentiation into IgA-secreting plasma cells, which subsequently migrate to mucosal surfaces to provide localized, antigen-specific immune protection through the production of secretory IgA (**Figure 1C**). Functioning as a critical filtration hub within the lymphatic circulatory system, LNs execute immune surveillance through their highly organized compartmental architecture. Afferent lymphatic vessels deliver tissue-derived antigens and professional APCs (particularly DCs) to the LNs, where T cell-mediated immune responses are initiated within the paracortical T cell zones, while follicular B cell activation and germinal center formation occur in the cortical regions. Specialized high endothelial venules within LNs facilitate lymphocyte recirculation, maintaining systemic immune cell homeostasis and ensuring rapid deployment of effector cells throughout the organism (54, 55). Complementing this network, the spleen, as the largest secondary lymphoid organ, serves as a central regulator of systemic immunity. Its distinctive microanatomical organization, comprising white pulp (supporting adaptive immunity) and red pulp (mediating innate immune functions), enables comprehensive immune surveillance. The marginal zone (MZ) of the spleen serves as the gateway for antigens and lymphocytes in the blood to enter the white pulp, where immune responses against blood-borne pathogens are initiated (55, 56) (**Figure 1D**). Through their distinct structural and functional properties, various organs meticulously regulate the development and functionality of immune cells, thereby enabling the host to mount effective responses against diverse pathogens and disease conditions.

**Figure 2:**
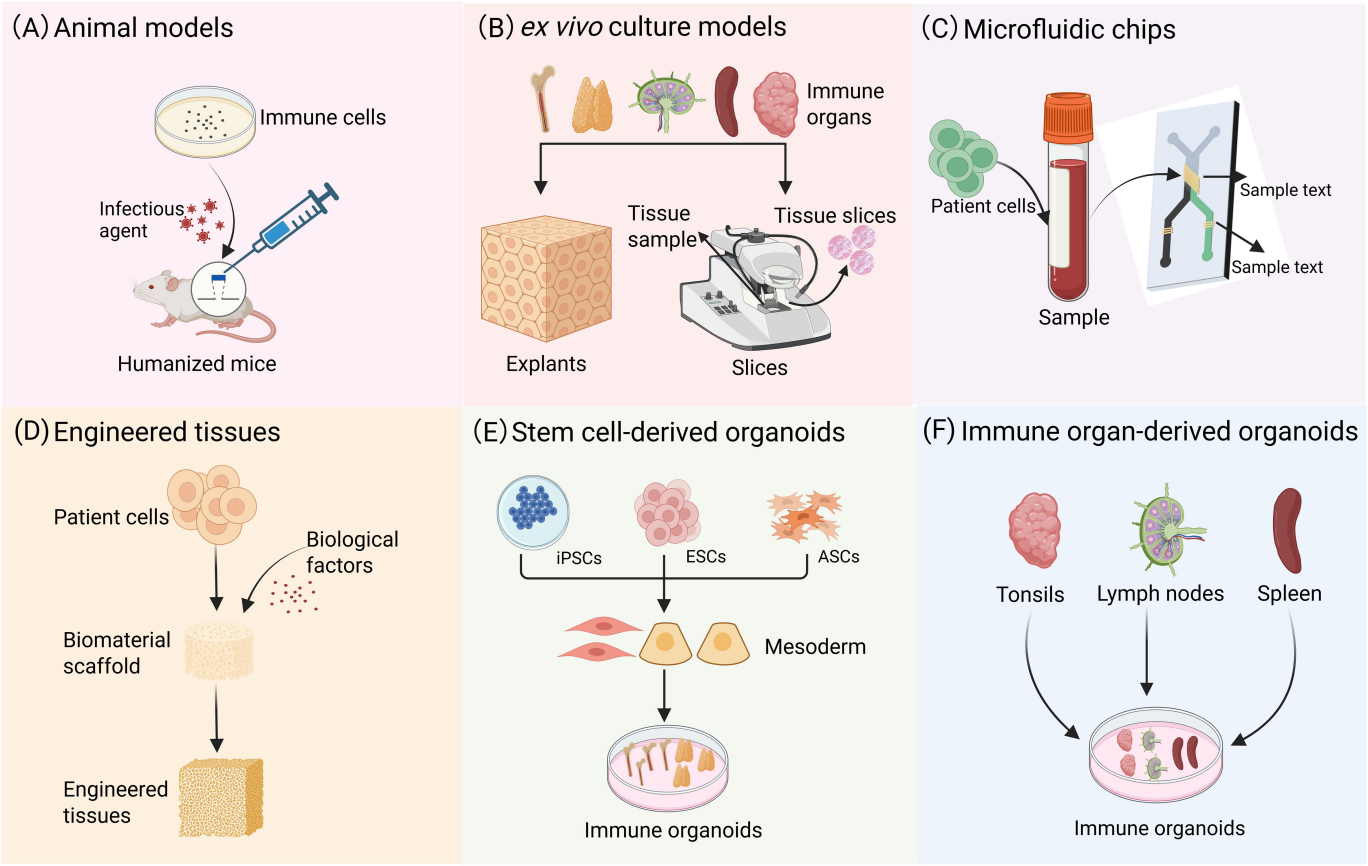
Modeling immune system: from animal models to organoids. **(A)** Animal models are capable of replicating comprehensive biological systems, including organ-organ interactions, the immune system, and the neuroendocrine system. **(B)** *In vitro* culture preserves the original cellular composition and spatial architecture of tissues, and this well-established technique is suitable for short-term studies. **(C)** Microfluidic chips can replicate complex microenvironments, such as blood flow and shear stress, and facilitate the simulation of interactions between different organs. **(D)** Engineered tissues provide a highly adaptable experimental platform for investigating the functions of diverse immune cells. **(E)** Tissue analogues with definite spatial structures formed through the 3D directional differentiation of ASCs or PSCs. **(F)** Organoid models can be derived from the direct culture of primary tissues, such as tonsils, spleen, and LNs.

**Figure 3:**
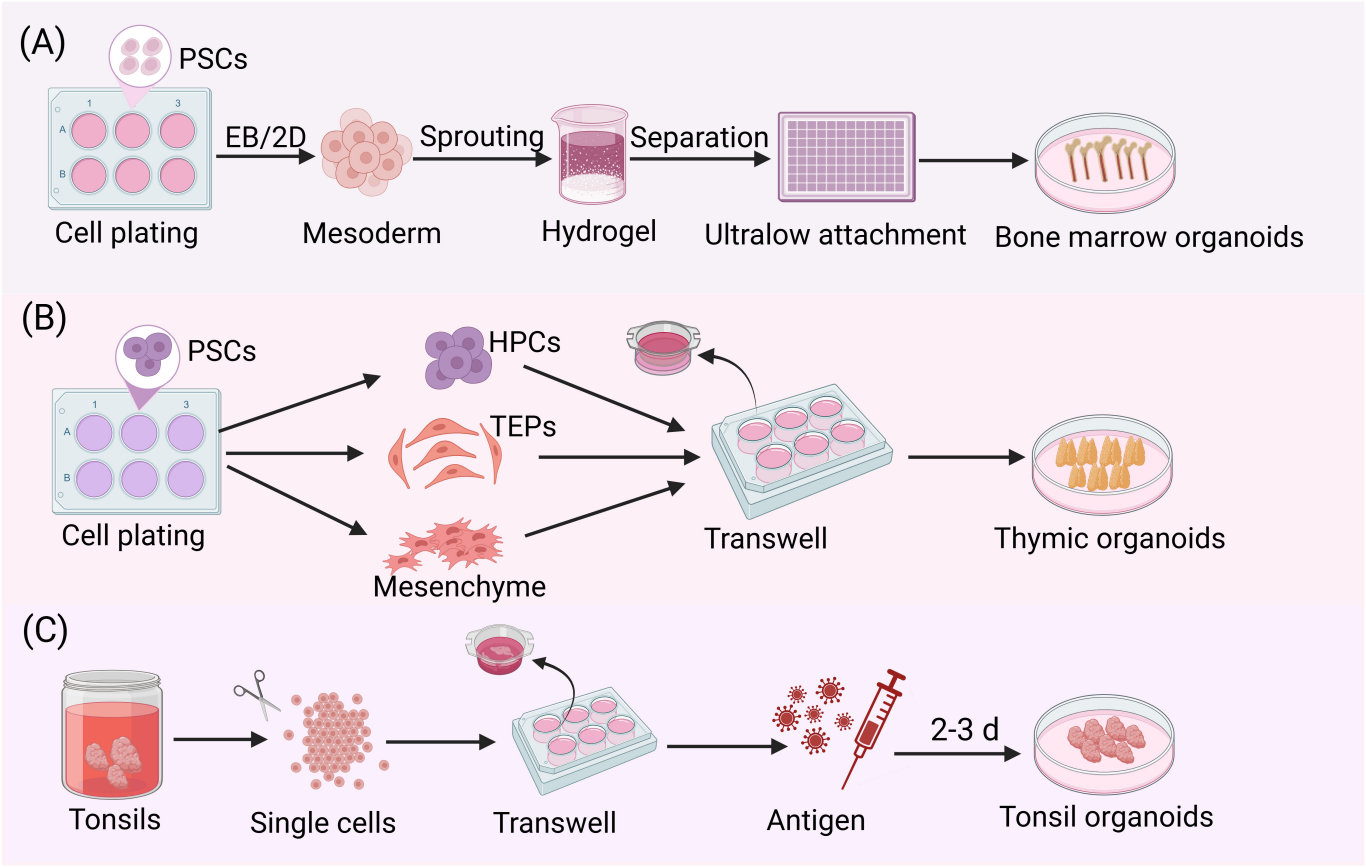
Stepwise protocols for culturing immune organoids. **(A)** After seeding iPSCs, mesodermal aggregates were formed either by direct addition of mesoderm induction medium or by first generating embryoid bodies followed by mesodermal differentiation. These aggregates were then embedded in Matrigel. Following the formation of vascular sprouts, individual sprouts were carefully isolated and cultured in 96-well ultralow attachment plate to ultimately generate bone marrow organoids. **(B)** hPSCs were plated and simultaneously differentiated into three critical progenitor populations: thymic epithelial progenitors (TEPs), HPCs and mesenchymal stromal cells. These cells were populations at an optimized ratio to generate stem cell-derived thymic organoids (sTOs) through 3D co-culture. Under air-liquid interface conditions, sTOs further differentiated into functionally competent TECs. **(C)** After mechanical dissociation of tonsil tissue into single-cell suspensions, the cells were co-seeded with the antigen of interest at high density on transwell inserts. Following several days in culture, tonsil organoids could be observed.

Many critical aspects of the lymphoid microenvironment, including dynamic cell migration and the regulatory mechanisms of stromal cells in T/B cell interactions, remain challenging to accurately model using conventional approaches. While humanized mouse models (**Figure 2A**) and 3D tumor organoids incorporating immune components provide partial research platforms, they fail to fully recapitulate the complex spatial architecture and functional characteristics of LOs. Recently developed *ex vivo* culture systems (**Figure 2B**), microfluidic chip technologies (**Figure 2C**), and engineered tissues (**Figure 2D**) have demonstrated advantages in simulating immune responses. However, these systems still cannot completely preserve the native multicellular composition of immune organs, precisely reconstruct tissue spatial organization, or faithfully reproduce the sophisticated regulatory networks of human adaptive immunity. In this context, immune organoid technology has emerged as a breakthrough solution. Through 3D self-organizing culture systems, it achieves functional reconstitution of stem cells or primary tissues. This advancement not only overcomes the technical limitations of existing models but also provides a revolutionary platform for studying immune cell interactions. More importantly, it opens new avenues for investigating adaptive immune mechanisms and developing translational applications.

## 3 Immune organoids: bridging the gap between traditional models and immune organs

Immune organoids can be categorized into strictly and broadly defined organoids based on their source and construction methodology (**Table 2**). Strictly defined organoids are spatially organized tissue analogs generated through the 3D differentiation of adult stem cells (ASCs) or pluripotent stem cells (PSCs), such as bone marrow and thymus organoids (**Figure 2E**). Their defining feature is stem cell-driven self-assembly and biomimetic tissue reconstruction. Broadly defined organoids generally refer to organoid models derived directly from primary tissues, such as spleen, LNs, and tonsils (**Figure 2F**). Notably, the formation of tonsil organoids does not rely on the differentiation of ASCs but is achieved through the spontaneous reorganization of tissue fragments. This characteristic makes them a unique model for studying tissue regeneration and the immune microenvironment. Below, we briefly outline the fundamental principles to consider when creating models for specific aspects of the immune system. We then summarize existing techniques and recent advances, with a focus on the advantages demonstrated by immune organoids.

**Table 2:** Characteristics of immune organoids.

Category	Pluripotent stem cells	Adult stem cells	Primary tissues
Advantages	Large-scale availability (57, 58)	Patient genetic heterogeneity capture, long-time culture expansion potential, media variability (57)	Native cellular composition (22)
Disadvantages	Impure cell culture, inability to capture genetic heterogeneity, ethical concerns (57, 58)	Incomplete tissue microenvironment representation (57)	Reduced proliferation capacity, limited lifespan (22)

### 3.1 Bone marrow organoids

As the central organ of the human hematopoietic system, the bone marrow plays a critical role in generating blood cells. Within this specialized tissue, HSCs exhibit remarkable characteristics, such as unlimited self-renewal capacity and the ability to continuously produce identical daughter cells, ensuring the ongoing hematopoietic process. Typically, HSCs reside in the G0 phase of the cell cycle, indicating a quiescent state where they are temporarily detached from active cell cycling. This quiescence endows HSCs with high differentiation potential, enabling them to rapidly activate and differentiate into various mature blood cells with specific functions—such as erythrocytes, leukocytes, and platelets—thereby maintaining the stability and normal function of the human blood system (59).

Most experimental work on bone marrow typically commences with cell suspensions acquired through bone marrow aspiration or biopsy. However, for immunohistochemical analysis, entire bone marrow samples are generally fixed (60). Although *in vitro* cultures of bone marrow have proven beneficial for investigating hematopoietic function, live slices and explants have not been extensively utilized for studying bone marrow immune function (61).

Current research on bone marrow-on-a-chip models primarily focuses on the development of biomimetic scaffolds and HSCs culture techniques, but there are still significant gaps in replicating the core biological functions of bone marrow—the hematopoietic differentiation process (62–65). Torizawa’s team pioneered the groundbreaking development of a biomimetic chip platform with comprehensive bone marrow microenvironment features, which innovatively integrates the cellular heterogeneity of bone marrow tissue and functional hematopoietic niches (65). However, it should be noted that their validation experiments were solely based on a murine model system, and the translatability of their findings to human bone marrow systems still requires rigorous experimental verification. To address this critical scientific challenge, the team achieved significant progress in 2020 by successfully constructing a vascularized human bone marrow biomimetic chip model (66). This model not only accurately recapitulates the dynamic features of hematopoietic processes but also effectively simulates pathological phenotypes of bone marrow dysfunction. In 2021, Nelson’s team achieved a technological breakthrough with the development of a high-throughput 96-well format bone marrow-on-a-chip system (67). The key design highlight lies in its simultaneous integration of microenvironmental features from critical hematopoietic niches, including the endosteal, central marrow, and perivascular regions. This sophisticated model exhibits not only remarkable architectural fidelity to native tissue organization, but more critically, establishes unprecedented physiological relevance at the functional level—most notably through its capacity to reliably sustain long-term *in vitro* expansion and maintain the self-renewal potential of primitive CD34^+^ HSCs.

Bone marrow engineered tissues modeling technologies have evolved from traditional static culture systems to dynamic biomimetic systems (68–70). The field primarily relies on static 3D culture systems using HSCs or mesenchymal stem cells, which have successfully validated immune cell generation. Notably, Mortera-Blanco’s team developed an innovative bone marrow biomimetic model using poly lactic-co-glycolic acid and polyurethane composite scaffold materials, achieving long-term expansion of cord blood mononuclear cells under cytokine-free conditions (69). Recent advancements have introduced bioreactor perfusion technology, enabling models to simulate physiological conditions in dynamic environments. For example, Nichol’s team successfully cultured HSCs in a rotating wall vessel bioreactor system using 3D polyacrylamide scaffolds with inverse opal crystal structures, where B lymphocyte differentiation was observed (70). These technological breakthroughs not only refine the bone marrow engineered tissues system but also provide new research platforms for investigating hematopoietic differentiation mechanisms and constructing blood disease models.

Current human *ex vivo* bone marrow models face significant technical limitations. A core issue is the lack of authentic sinusoidal endothelial structures, forcing many studies to rely on non-physiological human umbilical vein endothelial cells as substitutes (71). Although organ-on-a-chip technologies have made some progress in simulating certain bone marrow components, these systems still suffer from critical deficiencies. For example, there is an absence of functional stromal networks, and vascular formation is defective, leading to insufficient active hematopoiesis (66, 71–74). To overcome these technical barriers, Khan’s team pioneered the successful construction in 2022 of 3D bone marrow organoids containing mesenchymal stroma, myeloid cells, and sinusoidal vascular networks using human induced pluripotent stem cells (iPSCs) (75) (**Figure 3A**). This model accurately recapitulates key bone marrow components, including HSCs, myeloid cells, megakaryocytes, endothelial cells, and MSCs. Single-cell transcriptomic analysis confirmed the model’s high molecular-level similarity to native bone marrow tissue. Subsequently, Frenz-Wiessner’s team further developed more advanced *in vitro* models of the bone marrow microenvironment. By optimizing culture systems, they constructed complex organoids containing multilineage hematopoietic cells, functional mesenchymal stroma, and vascular networks, achieving high-fidelity simulation of key features of the bone marrow niche (26) (**Figure 3A**). However, current organoid models still face technical challenges, such as the absence of functional immune cells and insufficient vascular network maturation. These factors limit their ability to fully replicate the *in vivo* bone marrow microenvironment and represent key directions for future research breakthroughs.

### 3.2 Thymic organoids

As a vital lymphoid organ in the human body, the thymus plays an indispensable role in the adaptive immune response and serves as a specialized site for the generation and maturation of T lymphocytes (76). It modulates positive selection, negative selection, and tolerance establishment of T cells through interactions between its cortical and medullary regions, as well as with thymic epithelial cells (TECs) (77–79). Since no microfluidic models of the thymus have been identified to date, the following sections focus on models of *in vitro* cultures, engineered tissues, and thymic organoids.

The *in vitro* thymic culture system provides an important research platform for in-depth investigation of the key regulatory mechanisms of T cell development and the formation of central tolerance. Among these systems, the fetal thymus organ culture (FTOC) serves as a classical model that has played a foundational role in elucidating the microenvironmental requirements and molecular regulatory mechanisms underlying normal T cell development (32, 80–83). In recent years, Campinoti’s team has optimized the FTOC system and combined with whole-thymus perfusion technology to successfully obtain naturally decellularized extracellular matrix (ECM), achieving precise simulation and functional reconstruction of the thymic microenvironment (32). Meanwhile, the establishment of thymic tissue slice culture technology has enabled researchers to dynamically analyze the formation process of T cell tolerance under near-physiological conditions (84–88). Ross’s team utilized this technology to demonstrate that thymic precursors recognize self-peptides bound to the major histocompatibility complex, undergo positive and negative selection, and ultimately develop into a functional T cell repertoire with both immune responsiveness and self-tolerance, while systematically elucidating the molecular characteristics of each selection stage in thymic slices (87). Notably, with the advancement of research, thymic slice technology has been extended to human thymic tissue, providing new experimental approaches for exploring the unique features of human T cell development (89).

Successful thymic bioengineering models must precisely reconstruct key features of the thymic microenvironmental to support thymocyte maturation, positive selection, and terminal differentiation into functional single-positive CD4^+^ or CD8^+^ T cells, ultimately generating a diverse and self-tolerant T cell repertoire (90). The field of thymic tissue bioengineering remains in its nascent stages, with current research primarily focused on developing *in vitro* T-cell generation models. In a pioneering study, Poznansky’s team implanted murine thymic tissue fragments into porous cellular scaffolds. Upon achieving 80% confluence of stromal cells, they introduced human bone marrow-derived progenitor cells, demonstrating their efficient differentiation into functional T cells within 14 days (91). However, conventional *in vitro* T-cell differentiation systems (e.g., the OP9-DL1 co-culture system) still exhibit significant limitations in promoting terminal T-cell maturation and positive selection. Recent breakthroughs in human thymic modeling have led to the development of novel artificial thymic organoid systems that demonstrate remarkable robustness, effectively overcoming inherent limitations of animal models. Notably, researchers have successfully achieved complete differentiation from human HSCs to mature T cells under serum-free culture conditions (92). This advancement provides a transformative technological platform for both thymic development studies and T-cell-based immunotherapy. In 2024, thymic organoid research achieved significant breakthroughs. Scientists successfully established long-term expandable 3D TECs organoids from adult mouse thymic tissue and generated thymic organoids derived the stromal compartments of LOs (93, 94). Although the T-cell output levels in these bioengineered thymic models remain relatively low, potentially limiting in-depth analysis of the composition and functional diversity of the TCR repertoire, studies have shown that, where feasible, these models still exhibit a relatively broad coverage of TCR gene segments and comparable representative distributions to those observed in natural thymus tissue or blood samples (90, 92, 95).

While humanized mouse models hold significant value for immune system research and disease modeling, their inherent limitations cannot be overlooked: the absence of a physiological human thymic microenvironment prevents human T cells developed in mice from establishing functional interaction networks with other human immune cell subsets, thereby failing to reconstruct a complete human immune response. To overcome this bottleneck, researchers have focused on reconstructing the 3D thymic microenvironment *in vitro* to mimic its critical physiological functions (96, 97). In recent years, through pluripotent stem cell differentiation technology, scientists have successfully established transformation systems from human embryonic stem cells to functional TECs (98, 99). These culture systems can self-organize into thymus-like structures, supporting not only *in vitro* T cell generation but also maintaining functional activity after transplantation into immunodeficient mice. Notably, the team led by Stephan A. Ramos optimized the differentiation protocols for iPSCs into thymic epithelial progenitor cells and, by combining hematopoietic progenitor cells (HPCs) with mesenchymal cells in co-culture, successfully constructed the first functional homologous stem cell-derived thymic organoids (sTOs) *in vitro* (100) (**Figure 3B**). This advancement not only provides a powerful tool for investigating thymic development and mechanisms of T-cell differentiation but also establishes a critical foundation for development of novel therapies targeting immune-related diseases and strategies for thymus regeneration.

### 3.3 Spleen organoids

The spleen, as the largest secondary lymphoid organ in the body, performs a broad spectrum of immune functions while also plays a crucial role in hematopoiesis and red blood cell clearance (56, 101). Anatomically and functionally, the spleen is divided into the red pulp and the white pulp. In rodents, the interface between these two regions is designated as the MZ, whereas in humans, this transitional area is referred to as the perifollicular zone (102, 103). The red pulp houses macrophages, which primarily function to filter blood and recycle iron from senescent red blood cells. Structurally analogous to LNs, the white pulp comprises T-cell and B-cell regions (the latter also referred to as follicles) and is capable of mounting antigen-specific immune responses to safeguard the body against diseases caused by blood-borne bacterial, viral, and fungal pathogens (103). Moreover, the spleen plays crucial roles in modulating potentially harmful immune responses to the host. Despite advances in engineered tissues and stem cell research, engineered tissue models and stem cell-derived spleen organoids remain underexplored in this field. Therefore, the following sections mainly focus on *ex vivo* culture and microfluidic chips.

In the field of spleen research, traditional approaches have primarily relied on autopsy and animal models, but these methods fail to accurately replicate organ function under physiological conditions. As early as the 1970s, researchers attempted to develop mouse spleen slicing techniques, but these early methods were significantly limited in maintaining tissue viability and functionality due to technological constraints (104). With technological advancements, this field has achieved critical breakthroughs. James’s team pioneered the application of precision-cutting technology to human spleen modeling (105). This technique preserves the complete ECM structure and faithfully recreates the complex cellular interaction networks within the tissue microenvironment, providing a more accurate model for studying spleen function under both physiological and pathological conditions. Building upon this foundation, recent studies have further refined an innovative precision-cutting protocol for mouse spleen. By optimizing vibratome parameters and agarose embedding techniques, researchers can now efficiently prepare structurally intact and highly viable spleen slices suitable for organotypic culture for up to 48 hours (106). This technological breakthrough not only overcomes the limitations of traditional methods in maintaining tissue viability but also establishes a physiologically relevant experimental platform for spleen research.

Efforts to model the spleen on microfluidic devices have predominantly concentrated on its principal function of filtering red blood cells rather than its immunological role. A microengineering model termed the human spleen chip, developed by Rigat-Brugarolas’s team, effectively emulates the filtering function of the human spleen (107). This multi-layer microfluidic device was meticulously designed to replicate the microcirculation and physical characteristics of the spleen, including rapid closure and gradual reopening of microcirculation, a reticular structure with elevated hematocrit levels, and intercellular spaces between endothelial cells. The future incorporation of immune function components into these models may significantly enhance our capability to investigate the outcomes and mechanisms associated with blood-borne infections. In another study of microfluidic systems, researchers developed an oxygen-regulated spleen chip platform to simulate the splenic interendothelial gap (S-filter) and macrophage (M-filter) through two functional modules, S-Chip and M-Chip, respectively, to study the mechanical retention and phagocytosis processes of red blood cells under hypoxic conditions (108).

Engineered tissues has opened new avenues for spleen functional reconstruction and immunological research. Purwada’s team innovatively co-cultured primary mouse splenic B cells with transgenic 40LB stromal cells in a 3D system, successfully constructing a functional spleen organoid model (19, 20). This landmark achievement laid the foundation for subsequent human studies. In 2020, significant progress was made when scientists established a continuously proliferative human spleen organoid culture system by optimizing the processing protocols for human spleen samples (109). Most remarkably, these engineered human spleen organoids demonstrated remarkable therapeutic potential in transplantation experiments—when implanted into splenectomized mouse models, they self-assembled into spleen-like tissue structures and effectively restored the host’s erythrocyte clearance function within 4 weeks post-transplantation. These findings validate the feasibility of engineered tissues spleen and advance its clinical translation.

### 3.4 Lymph nodes organoids

The LN is structurally divided into three primary regions: the cortex, paracortex, and medulla. The cortex, as the outermost layer of the LN, primarily houses B cell follicles, which are niches enriched with B cells and follicular DCs, along with the interfollicular zone that delineates these follicles. The paracortex, an internal region also known as the T-cell zone, is predominantly occupied by fibroblastic reticular cells forming a ductal network throughout this area. Proximal to the efferent lymphatic vessels, the medulla contains the medullary sinuses (110). LNs are critical components of the lymphatic and immune systems, playing a pivotal role in detecting, responding to, and eliminating harmful substances efficiently (111). Developing a model of LNs could significantly enhance our understanding of the mechanisms underlying immune response generation.

As crucial immune organs, LNs exhibit intricate cellular interaction networks, dynamic lymphocyte migration, and antigen recognition mechanisms, which are primarily studied using live imaging techniques (112–114). To investigate the deep tissue architecture of LNs, vibratome-sectioned live slices with thicknesses ranging from 250 to 400 μm can be prepared for real-time imaging monitoring (115, 116). This tissue slice platform enables temporal visualization of individual cells while simultaneously monitoring global changes in surface marker expression and quantifying bulk cytokine secretion from intact slices or explants to assess immune responses (29, 117, 118).

Microfluidic chip technology has been effectively utilized to emulate the intricate environment and functionality of LNs. This technology not only integrates seamlessly with precision slice culture techniques but also establishes an experimental platform that more closely mimics *in vivo* physiological conditions for cell research. Moreover, it offers robust technical support for elucidating the interaction mechanisms between cells and the precise regulation of cellular behavior. Ross’s team employed microfluidic integrated optical imaging to investigate cytokine diffusion within LNs, thereby quantifying the diffusion of bioactive molecules in living tissues (119). In a study conducted by Moura Rosa’s team, a microfluidic device was utilized to investigate the interaction between infused T cells and adherent DC cells, as well as the effects of varying shear stresses within the device (120). To date, LN on-chip models have only partially recapitulated select features of human LNs (121).

Unlike other engineering systems that focus on tissue architecture, engineered tissues for immunity research predominantly aim to replicate specific immune functions (16). Giese’s team developed a novel *in vitro* model of human LNs, which can be maintained continuously for several weeks (122). This extended operational period enables prolonged and repeated drug exposures, facilitating the induction and monitoring of both cellular and humoral immune responses. Tomei’s team encapsulated FRCs within macroporous polyurethane scaffolds composed of type I collagen and Matrigel (123). They applied a gap flow ranging from 1 to 23 μL/min in this *in vitro* 3D system. This experimental setup successfully recapitulates the *in vivo* morphology of FRCs and demonstrates the critical role of lymphatic flow in LN function. The results demonstrate that lymphatic flow significantly enhances the organization of FRCs, whereas FRCs in the absence of flow do not produce CCL21.

### 3.5 Tonsil organoids

The palatine tonsils, as MALT located in the upper respiratory tract, together with the adenoids and lingual tonsils, form part of the Waldeyer’s ring, which constitutes the first line of immune defense against inhaled or ingested pathogens (51, 52). As prominent components of the Waldeyer’s ring, tonsils and adenoids are functionally closely associated with nasopharynx-associated lymphoid tissues in rodents and other species. The cellular architecture of these tissues is intricate, featuring germinal centers within B-cell follicles and T-cell-rich regions, closely resembling that of LNs. Notably, the tonsil lacks afferent lymphatic vessels, enabling direct contact with antigens from the external environment. Importantly, the tonsil plays a crucial role in inducing B cell immune responses, particularly following direct antigen stimulation (124, 125). Although tonsil tissue is relatively accessible, research on engineered tissues applications remains at an exploratory stage. To date, no studies have reported the successful generation of stem cell-derived tonsillar organoids. Therefore, the following sections will primarily focus on research progress regarding broadly defined organoids.

Currently, research on tonsils primarily utilizes live slices or explant culture techniques. Studies have shown that human tonsil tissue blocks can be effectively maintained *in vitro* for up to four days, providing an important model for investigating the dynamic behavior of immune cells within their microenvironment (126). Additionally, Grevel’s team employed a tonsil explant culture model to deeply analyze the molecular mechanisms of host cell interactions and their interplay with pathogens (118). These studies confirm that this *in vitro* culture system effectively supports comprehensive research on microbiome characteristics, histopathological properties, and viral susceptibility of patient-derived tonsil tissues (127, 128).

The *in vitro* culture of tonsil sections suffers from inherent limitations, such as short preservation time and high contamination risk. These limitations severely restrict its applications. Wagar’s team innovatively developed a tonsil organoid model based on high-density culture on low-attachment surfaces. By maintaining lymphoid tissue cells derived from tonsils, spleen, and LNs, this model successfully preserves the structural and functional characteristics of the original tissue (22) (**Figure 3C**). This system can effectively induce B cells to undergo SHM, class switch recombination, and affinity maturation while producing specific antibodies, providing a controlled, high-throughput, and cost-effective platform for studying adaptive immune responses. However, the model still exhibits significant limitations: first, its reliance on spontaneous cell aggregation leads to poor reproducibility in organoid size and morphology, severely impacting its application in vaccine and drug screening. Second, whether the model contains critical lymphoid stromal cells remains unclear, potentially compromising the simulation of a complete immune microenvironment.

## 4 Application and perspectives of immune organoids in immunological research

### 4.1 Current applications of immune organoids

**Figure 4:**
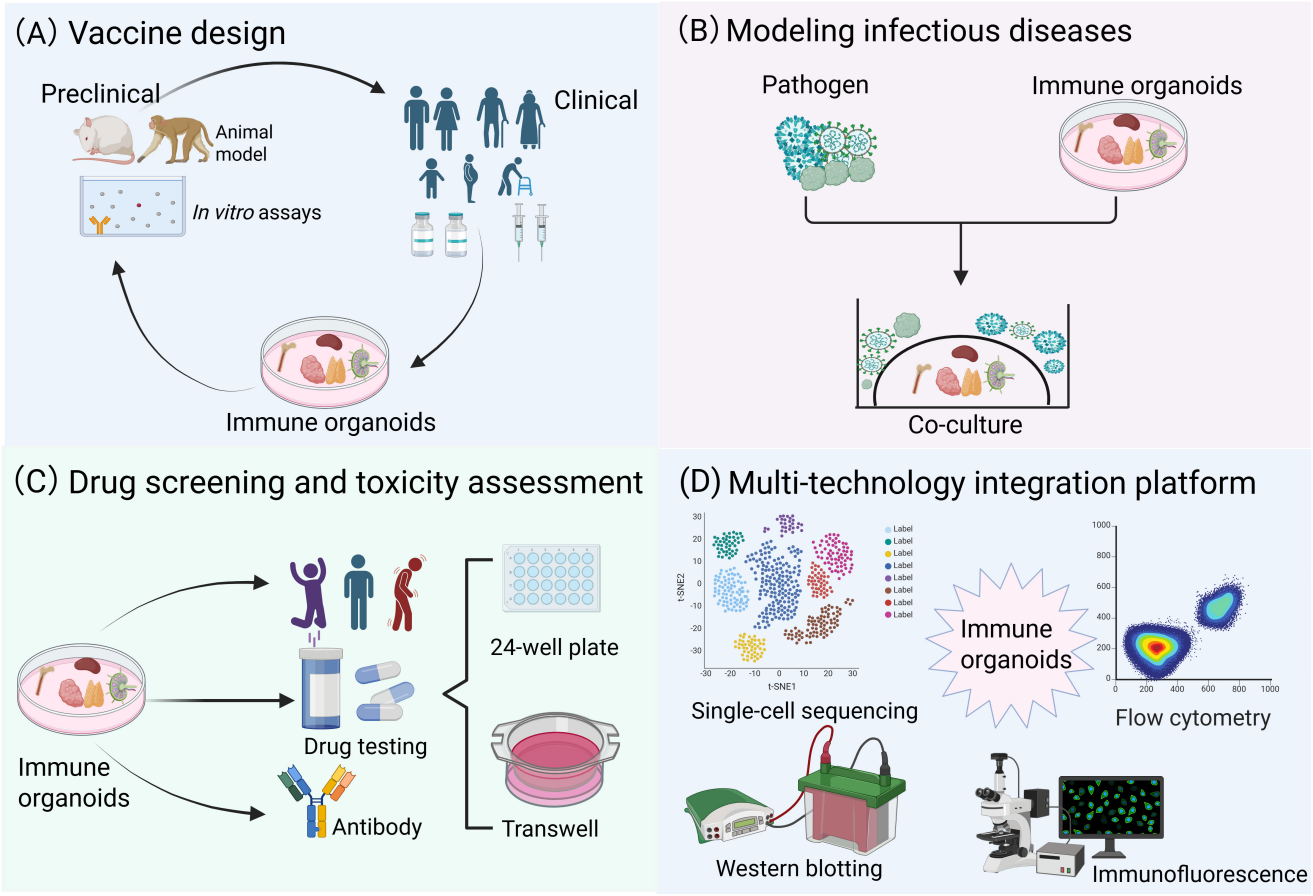
Multidisciplinary convergence platforms: translational applications in biomedical research. **(A)** Immune organoids can be rationally designed for vaccine development, enabling a more precise simulation of the human immune response mechanism and supporting the evaluation of vaccine immunogenicity and safety. **(B)** Immune organoids co-culture systems for modeling infectious diseases and cancer. **(C)** Immune organoids exhibit significant potential in drug screening and toxicity assessment. **(D)** Immune organoids are compatible with many traditional and cutting-edge technologies.

In vaccine development (**Figure 4A**), previous studies have attempted to simulate vaccine-induced adaptive immune responses using organ-on-a-chip systems (13), synthetic biomaterial-based immune organoids (129, 130), or LN tissue slices (117), however, all these systems exhibit significant limitations. A research team utilized the high-throughput advantage of tonsil organoids to systematically compare the differences in immune responses induced by inactivated vaccines, live attenuated vaccines, and wild-type viruses, thereby providing critical technical support for investigations into vaccine mechanisms (22, 131). In disease modeling (**Figure 4B**), lymphoma organoids and bone marrow organoids (75, 132), as novel *in vitro* models, have demonstrated significant value in hematological disease research. Lymphoma organoids can stably maintain tumor genetic characteristics and interactions within the immune microenvironment, offering a reliable platform for studying the pathogenesis of follicular lymphoma and evaluating drug efficacy. Bone marrow organoids, in contrast, accurately mimic the physiological and pathological states of the bone marrow microenvironment, making them suitable for studying diseases such as leukemia and myelofibrosis. By incorporating patient-derived cells or specific factors (e.g., TGF-β), these models can reconstruct disease microenvironment features, thus serving as ideal tools for mechanistic exploration and therapeutic optimization. In drug screening and toxicity assessment (**Figure 4C**), patient-derived lymphoid organoid models retain patient-specific tumor microenvironments, enabling precise evaluation of immunotherapies effects (e.g., bispecific antibodies) on tumor cell killing and the activation properties of T-cell. Bone marrow organoids can systematically assess the toxic effects of drugs on hematopoietic function, offering reliable preclinical safety data for evaluating the myelosuppressive risks of chemotherapy and targeted therapies (75, 132). Notably, immune organoids are compatible with many traditional and cutting-edge technologies (**Figure 4D**). For instance, in the case of tonsil organoids (22), single-cell RNA sequencing (scRNA-seq) successfully identified germinal center-like B cells (CD38^+^/CD27^+^), while transcriptomic profiling confirmed stable gene expression patterns across different culture durations. Flow cytometry analysis precisely quantified shifts in immune cell populations following stimulation with live-attenuated influenza vaccine. Notably, ELISA assays detected influenza-specific IgG secretion as early as 7 days post-vaccination, demonstrating the functional maturation of naive B cells into hemagglutinin-specific antibody producers.

### 4.2 Future challenges and prospects of immune organoids

While organoid technology has made remarkable progress, the lack of standardized protocols continues to limit experimental reproducibility and consistency. Notably, China recently issued its first national standard for organ-on-a-chip technology — “General Technical Requirements for Skin-on-a-Chip” (GB/T 44831-2024), which sets a crucial benchmark for quality control in this field (133). Furthermore, disruptive technologies like 3D bioprinting and automation are bringing revolutionary opportunities to organoid research. 3D bioprinting platforms can efficiently construct complex structures such as liver organoids and lung cancer organoids (134, 135), with researchers having successfully simulated liver lobule-like structures and alveolar tissues *in vitro*. The automated microfluidic platform developed by Tay’s team has enabled high-throughput dynamic drug screening using pancreatic tumor organoids (136). The application of gene-editing tools like CRISPR/Cas9 has further facilitated genotype-phenotype relationship studies in organoids (137). Regrettably, these cutting-edge technologies remain largely unexplored in the field of immune organoids. Looking forward, immune organoid research urgently requires breakthroughs in several key areas: first, standardized culture systems and evaluation metrics must be established to enhance experimental reproducibility. Second, integration of microfluidics and 3D printing technologies is essential to construct more physiologically relevant immune microenvironments. Most importantly, comprehensive utilization of single-cell sequencing, spatial transcriptomics and other omics technologies will be essential to deeply analyze the dynamic regulatory mechanisms underlying immune responses. Only through the interdisciplinary convergence of multiple technologies can immune organoids truly evolve into an ideal platform for vaccine development, tumor immunotherapy, and autoimmune disease research.

## 5 Concluding remarks

In recent years, significant advancements have been achieved in the development and application of *in vitro* and *ex vivo* immune models. This review highlights several experimental systems utilized to investigate and emulate immune interactions, including *ex vivo* culture systems, organ-on-a-chip platforms, engineered tissue technologies, and stem cell-derived organoids. These technological platforms demonstrate unique value in adaptive immunity research and clinical translation applications (such as vaccine development) by integrating engineering advancements with immunological theories. Notably, these systems exhibit remarkable complementarity in functionality and application, with the tissue slice culture system combining engineered tissues with microfluidic technology being particularly outstanding. This multidisciplinary integrated platform can more accurately simulate the complex immune interaction networks observed *in vivo*, providing novel technological approaches for immunological research. Looking ahead, the integration of immune organoids with other organoid systems will open new research directions. For instance, coupling tonsil organoids with airway mucosal organoids could simulate respiratory mucosal immune responses, while combining LN organoids with intestinal organoids may help elucidate the regulatory mechanisms of gut-associated lymphoid tissue. Although current organoid technology still faces challenges related to functional completeness and technical standardization, its future development will undoubtedly break through the limitations of single-model paradigms, driving immunological research to new heights through multi-system integration.
